# Amyotrophic lateral sclerosis modifies progenitor neural proliferation in adult classic neurogenic brain niches

**DOI:** 10.1186/s12883-017-0956-5

**Published:** 2017-09-06

**Authors:** Lucía Galán, Ulises Gómez-Pinedo, Antonio Guerrero, Jose Manuel García-Verdugo, Jorge Matías-Guiu

**Affiliations:** 1 Amyotrophic Lateral Sclerosis Unit, Department of Neurology, Hospital Clínico San Carlos, Calle Profesor Martín Lagos s/n, 28040 Madrid, Spain; 2 Institute of Neurosciences, Hospital Clínico San Carlos, Madrid, Spain; 3 Cavanilles Institute of Biodiversity and Evolutionary Biology, Comparative Neurobiology Unit, Universidad de Valencia, Paterna, Spain

**Keywords:** Adult neurogenesis, Amyotrophic lateral sclerosis, Frontotemporal dementia, TDP-43, Neurodegenerative diseases

## Abstract

**Background:**

Adult neurogenesis persists through life at least in classic neurogenic niches. Neurogenesis has been previously described as reduced in neurodegenerative diseases. There is not much knowledge about is adult neurogenesis is or not modified in amyotrophy lateral sclerosis (ALS). All previous publications has studied the ALS SOD1 (superoxide dismutase) transgenic mouse model. The purpose of this study is to examine the process of adult neurogenesis in classic niches (subventricular zone [SVZ] and subgranular zone [SGZ] of the dentate gyrus) in patients with amyotrophic lateral sclerosis (ALS), both with (ALS-FTD) and without associated frontotemporal dementia (FTD).

**Methods:**

We studied 9 autopsies of patients with ALS (including 2 with ALS-FTD) and 4 controls. ALS was confirmed histologically. Studies of the SVZ and SGZ were conducted using markers of proliferation (Ki-67, PCNA), of pluripotent neural progenitor cells (GFAPδ), neuroblasts (PSA-NCAM, DCX, TUJ1), and an astrocyte marker (GFAP). Results were analyzed with non-parametric tests. We then studied correlations between the different markers and the percentage of phosphorylated TDP-43 (pTDP-43).

**Results:**

We observed a statistically significant increase in proliferation in the SVZ in all patients with ALS. While this increase was more marked in ALS forms associated with dementia, the small sample size does not permit a statistical subgroup analysis. In contrast, proliferation in the SGZ was decreased in all patients. These alterations showed a positive and direct correlation with the percentage of pTDP-43 in the SVZ, and a negative, exponential correlation with that percentage in the SGZ.

**Conclusions:**

We observed alterations of the proliferation of neural progenitor in classic adult neurogenic niches in patients with ALS. The 2 neurogenic niches exhibited opposite changes such that proliferation increased in the SVZ and decreased in the SGZ.

**Electronic supplementary material:**

The online version of this article (10.1186/s12883-017-0956-5) contains supplementary material, which is available to authorized users.

## Background

Adult neurogenesis was first described in animal studies in the 1960s [1]. It was later observed in adult primates, including humans [2]. Two well-known regions of the adult brain are widely held to be neurogenic zones: the subgranular zone of the hippocampal dentate gyrus (SGZ) [3] and the subventricular zone (SVZ) [4]. Other zones in the brain may also act as neurogenic niches, although this may only occur in certain conditions [5, 6].

The exact purpose of maintaining neurogenesis throughout adult life is unknown. Neurogenesis in the SGZ has been linked to the consolidation of memory [7]. In rodents, cells from the SVZ migrate from the rostral migratory stream (RMS) to the olfactory bulb. Based on this finding, scientists believe that the SVZ plays a role in the sense of smell. Nevertheless, the role of SVZ neurogenesis in humans, who may lack a true RMS, is not so clear [8]. On the other hand, when specific pathological conditions are present, the SVZ seems to be able to increase proliferation and modify cell migration; its potential restorative function has been observed following stroke [9].

Different studies have pointed to altered neurogenesis in neurodegenerative diseases [6, 10]. Most of them have found decreases in neurogenesis in these diseases, but findings vary depending on whether studies were carried out in humans or animals, the niche studied, and the moment in the course of the disease [10]. Amyotrophic lateral sclerosis (ALS) is a rapidly progressing neurodegenerative disease of unknown etiology. At present, there are no curative treatments [11]. Although ALS was previously thought to impair only motor neurons, we now know that it affects other cells and functions within the nervous system [11, 12]. Phosphorylated TDP-43 (pTDP-43) is a pathologic lesion characteristic of some degenerative diseases as ALS and frontotemporal dementia (FTD), but also inclusion body myositis [13–16] The only approved treatment, riluzole, is only moderately effective [17]. Few studies have examined the state of adult neurogenesis in ALS, and almost all of them are in animal models [18–21]. The only published human study is a report on a single case, and the same case now forms part of the series presented here [21]. This study analyses adult neurogenesis in classic niches in patients with ALS.

## Methods

### Patients

We analyzed the brains of 9 ALS patients (including 2 with FTD) and 4 controls (patients with no history of neurodegenerative disease who died in our hospital).

Patients’ medical records were reviewed to compile the variables related to the disease (age at onset, sex, time until diagnosis, time until death, form at onset, associated diseases, neuroimaging findings and presence of neuropsychiatric symptoms). We also checked the control group’s medical records to ensure absence of neurodegenerative disease.

### Tissue processing

Brains from patients and controls were sectioned in coronal slices 1 cm thick from the frontal to the occipital areas. All specimens were encased in paraffin and subsequently sliced into 6 μm sections with a microtome. Tissue sections were deparaffinized and washed in 0.1 M PBS. Epitopes were unmasked in a 10 mM sodium citrate buffer with a pH 6 at 96 °C for 20 min.

The immunohistochemical study analyzed the medial/central part of the SVZ and the SGZ of the dentate gyrus. Tissues were washed in PBS and then incubated for 1 h in blocking solution (PBS, 0.2%, Triton 10%, normal goat serum). They were subsequently incubated for 24 h with primary antibodies (Additional file 1: Table S1) diluted with PBS. After incubation with the primary antibody, the tissue sections were thoroughly washed in PBS before being incubated with the appropriate Alexa-Fluor antibody during 24 h (Additional file 1: Table S1). After sections had been washed, they were mounted in ProLong Gold reagent with DAPI (Molecular Probes, Invitrogen).

The quantitative immunohistochemical study of pTDP-43 focused on neurons in the dentate gyrus.

We analyzed 10 different fields for each of the antibodies under study; the result given is the mean of all 10 measurements. Results are expressed as cells positive for each antibody per 500 μm^2^.

For the quantitative study, we selected only those samples of the SVZ and SGZ in which both cells and cell layers remained intact.

All quantifications have been made for two differents investigators that were blinded for the patients diagnostic.

### Statistical analysis

Data are expressed as mean ± SD. Parametric tests could not be used owing to our small patient sample. Instead, we compared means using the Mann-Whitney U test. Statistical significance was set at *p* < 0.05. This test was not used for subgroups with fewer than 3 individuals. Since sample sizes were small, no multivariate tests were performed. We compared controls to all ALS patients, and controls to ALS patients subgroup without dementia, for all study variables. Since there were only 2 patients with frontotemporal dementia, that group was not included in the comparative analysis. We used the Pearson test to check for presence or absence of any correlations between presence of pTDP-43 and neurogenesis. Statistical analysis was performed using Prism© software. Graphs of the results were also created with Prism©.

## Results

### Clinical data

We studied 9 patients with ALS (2 had associated FTD) and 4 controls.

Mean age at time of death was 65.60 ± 15.94 for patients and 69.50 ± 11.38 for controls. Age differences between cases and controls were not significant. Fifty-five percent of the patients were men, as were 75% of the controls.

Regarding patient characteristics, form of onset was bulbar in 55% vs 21% in our historical series (unpublished data). Mean age at time of diagnosis was 64.33 ± 16.75 years (62.33 ± 11.2 in our historical series). Mean survival of patients after diagnosis was 12.44 ± 17.22 years (35.47 ± 17.21 in our historical series); survival after symptom onset was 24.88 ± 19.34 years (45.87 ± 24.12 in our historical series). Dementia was present in 2 patients (22%) vs 8% in our historical series. None of the patients in this study had a family history of ALS, whereas 8.9% of those in the historical study did. Regarding treatment, 3 patients (33%) were not being treated with riluzole when they died (32% in our historical series).

The two patients with FTD have first developed the cognitive symptoms and being diagnosed of FTD in our dementia clinic and then some years after they have developed the motor neuron symptoms. One of the patients have first begun with language alteration and then behavior problems and the other one with behavior problems, memory problems were developed lately. Regarding the motor neuron symptoms they both have developed first bulbar symptoms with few clinical spinal disease.

The mean time between death and autopsy was 5 ± 2 h. The mass of the fixed brains was 1210 ± 147.00 g for patients and 1290 ± 72.73 g for controls (differences not significant).

A summary of the clinical data is provided in the supplemental material (Additional file 1: Table S2). Amyotrophic lateral sclerosis was confirmed in all patients by an anatomical pathology study including pTDP-43 immunohistochemistry (Additional file 1: Table S3).

### Structural organization of the SVZ in patients with ALS

**Fig. 1:**
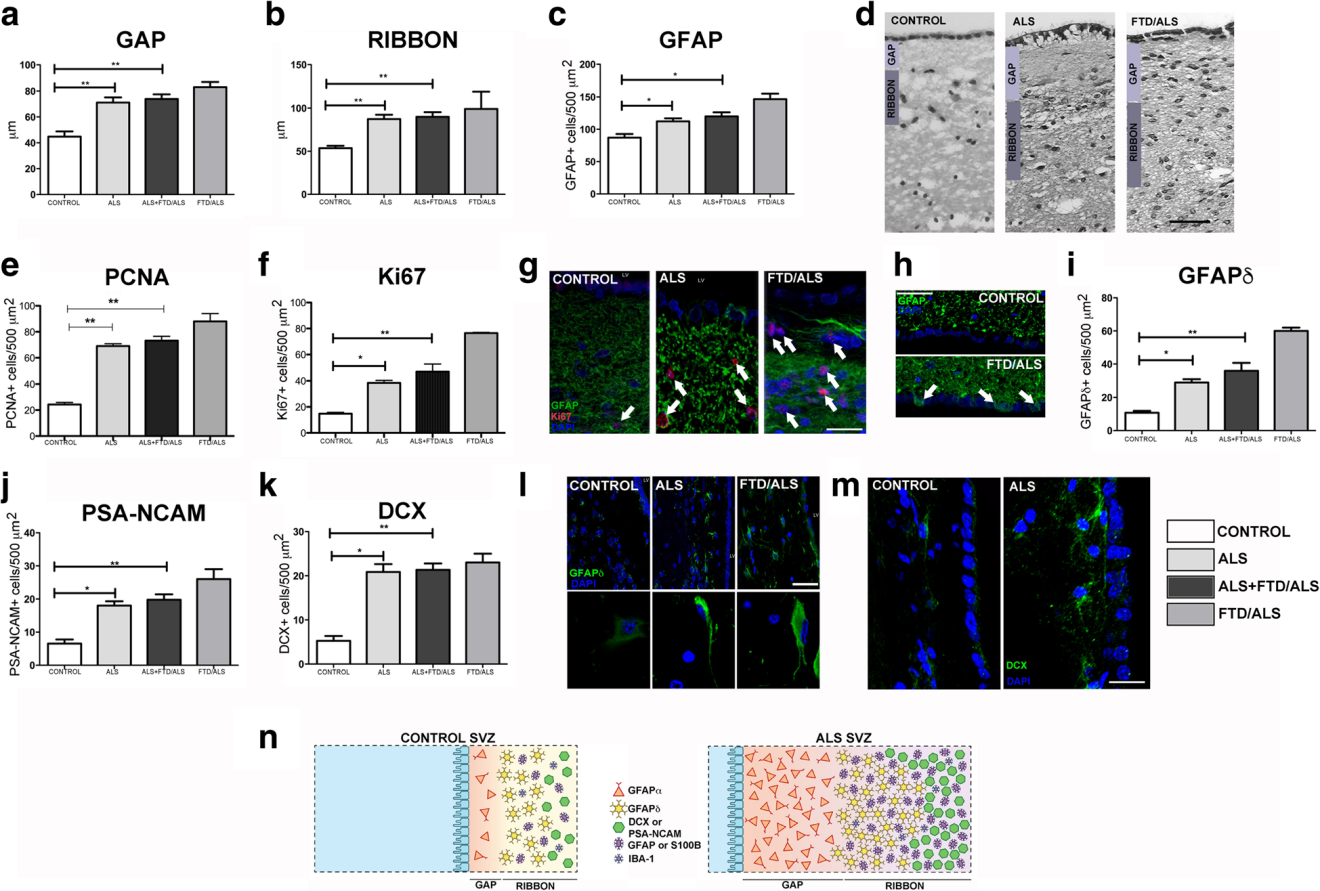
Changes in SVZ morphology in patients with ALS. **a**-**b** Illustrations show increases in thickness of the gap and ribbon layers; increases were greater in patients with ALS-FTD. **c** Increased expression of GFAP in the gap and ribbon layers in patients with ALS and ALS-FTD. **d** Images from the semithin sections of the SVZ showing the ventricular wall (V), the ependymal monolayer (I), the gap layer (II), the ribbon layer (III) and the parenchyma (IV). Data from these images was used to prepare the graphics in A and B. **e**-**f** Quantitative graphics representing the proliferative activity in the SVZ of ALS patients based on the analysis of the cell proliferation markers PCNA and Ki-67. PCNA and Ki-67 showed significant increases in PCNA and Ki-67 marking. **g** Confocal microscopy images of the SVZ in controls and patients with ALS and ALS-FTD showing increased Ki-67 and GFAP marking in patients, arrows show a immunopositive cells. **h** Images of SVZ of Control and ALS/FTD Patients. IN ALS/FTD, a displayed GFAP positive projections in contact with the ventricle (arrows). In control samples no were observed. **i** Graph displaying expression of GFAPδ in the SVZ of healthy individuals and patients with ALS and ALS-FTD; expression is significantly higher in patients. **j**-**k** Graphs showing increased expression of the proteins occurring in neurogenesis and neuronal migration in the adult central nervous system (doublecortin, PSA-NCAM); protein levels were significantly higher in patients with ALS or ALS-FTD than in healthy controls. **l** Immunofluorescence study of GFAPδ expression showing increased levels in the SVZ in ALS or ALS-FTD patients. In the details it is observed that in patients greater immunohitochemistry marking shown. **m** Images showing cells positive for doublecortin near the lateral wall. Their elongated fusiform shape is typical of migrating cells (arrows). **n** Illustration of typical SVZ morphology from controls and ALS patients, showing the main changes we observed: thickening of the gap and ribbon layers with increased expression of markers of astrocytes, cell proliferation, and neurogenesis. Graphs show the mean + standard error (*p < 0.05; **p < 0.01). Scale bars: D: 50 μm; G-H, L and M: 15 μm

In humans, the architecture of the SVZ in the lateral wall of the ventricle includes 3 layers. The first is a monolayer of cells in contact with the ventricle (ependymal layer), followed by a hypocellular gap and an additional layer comprising mainly astrocytes (ribbon) [4]. Since the SVZ may vary in size and composition according to the region analysed [4], this study focused on its body. While there were no differences between patients and controls in the size of the ependymal layer, there was a significant increase in the thickness of the gap layer in ALS patients (Additional file 1: Tables S4 and S6; Fig. 1a and d). Mean thickness of the gap layer in controls was 44.75 ± 8.02 μm vs 73.78 ± 3.57 in ALS patients (*p* < 0.01). This difference was also present for patients with ALS without dementia: 71.14 ± 3.99 μm (*p* < 0.01). Patients with dementia exhibited a thicker layer (83 ± 5.66 μm), but this subgroup was so small that a statistical analysis could not be performed. This increase in size of gap layer was due to abundant cell bodies structurally similar to astrocytes with numerous intermediate filaments. In addition, we observed a marked increase in the thickness of the ribbon (Additional file 1: Tables S4 and S6; Fig. 1b and d). Ribbon thickness in controls was 53.5 ± 5.51 μm vs 89.89 ± 16.09 μm in ALS patients (*p* < 0.005). The difference between controls and patients with ALS and no dementia was also significant at 87.29 ± 13.28 μm (*p* < 0.01). As in the case of the gap layer, the increase in ribbon thickness also appeared to be greater in patients with dementia (99 ± 28.28 μm).

### Proliferation of neural progenitor cells in the SVZ

Proliferative neural progenitor cells in the SVZ were studied using PCNA and Ki-67 [21, 22].

For PCNA, the mean number of marked cells in controls was 24.25 ± 3.30 cells/500 μm^2^ vs 73.22 ± 9.83 cells/500 μm^2^ in all ALS patients (*p* < 0.005). This difference was also present in patients with ALS without dementia: 69 ± 1.83 cells/500 μm^2^ (*p* < 0.01). It also seems to be bigger in ALS-FTD patients: 88 ± 8.49 cells/500 μm^2^ (Additional file 1: Tables S4 and S6; Fig. 1e).

For Ki-67, the mean number of marked cells in controls was 14.75 ± 2.06 cells/500μm^2^ vs 46.89 ± 17.40 cells/500 μm^2^ for all patients with ALS (*p* < 0.01). This difference was also observed in cases of ALS without dementia: 38.43 ± 1.99 cells/500 μm^2^ (*p* < 0.05). As with PCNA, the difference in the number of marked cells seems to be even greater in patients with dementia: 76.5 ± 0.75 cells/500 μm^2^ (Additional file 1: Tables S4 and S6; Fig. 1f and h).

The marker GFAPα was also analyzed. Although it is an astrocyte marker, the glial characteristics of proliferative neural progenitor cells mean that they may be signaled by GFAPα, provided that other proliferation markers co-localize [20]. This marker also delivered higher numbers of marked cells in patients. Cell count for controls was 87 ± 11.34 cells/500 μm^2^ vs 119.9 ± 18.97 cells/500 μm^2^ in all ALS patients (*p* < 0.05), 112.3 ± 4.66 cells/500 μm^2^ in ALS patients without dementia (*p* < 0.05), and 146.5 ± 12.02 cells/500 μm^2^ in FTD-ALS patients (Additional file 1: Tables S4 and S6; Fig. 1c). Given that cells marked with GFAPα were also indicated by other proliferation markers (Fig. 1g), at least some of these cells correspond to proliferative neural progenitor cells.

Cases with ALS-FTD also displayed cells with processes extending through the ependymal layer to reach the lateral ventricular wall (Fig. 1h). These cells had previously been described in mice in conditions entailing increased neurogenesis [23], in human they had been only described in one of the cases with FTD previously published [21].

### Proliferation of pluripotent neural cells (PNCs) in the SVZ

We used the GFAPδ marker to study PNCs [24–27]. This marker revealed a significantly higher number of these cells in all ALS patients (35.89 ± 14.49 cells/500 μm^2)^ compared to controls (10.75 ± 2.36 cells/500 μm^2^)). The ALS subgroup without dementia also differed significantly from the controls, with 29 ± 2.05 cells/500 μm^2^; (*p* = 0.01). The difference between controls and patients with both ALS and dementia appeared to be even greater: 60 ± 2.83 cells/500 μm^2^ in the ALS-FTD subgroup. However, a statistical analysis could not be performed due to the small sample size (Additional file 1: Tables S4 and S6; Fig. 1i and l). GFAPδ staining was stronger in ALS patient than in controls and cells has a fusiform morphology.

### Proliferation of neuroblasts in the SVZ

Neuroblasts were studied with PSA-NCAM, doublecortin, and Tuj-1 [9, 28–31]. Using PSA-NCAM, we observed more numerous marked cells in all ALS cases than in controls: 6.5 ± 2.51 cells/500 μm^2^ in controls vs 19.78 ± 1.63 cells/500 μm^2^ in the ALS total (*p* < 0.01). A significant difference was also present between controls and ALS patients without dementia at 18 ± 3.51 cells/500 μm^2^ (*p* = 0.01), and this may be even greater for cases of ALS-FTD at 26 ± 3 cells/500 μm^2^. Nevertheless, the low number of cases does not allow us to determine the statistical significance of this result (Additional file 1: Tables S4 and S6; Fig. 1j).

Using doublecortin (DCX), we found a significantly higher number of marked cells in the ALS patient than in controls: 5.25 ± 2.22 cells/500 μm^2^ in controls vs 21.33 ± 1.44 cells/500 μm^2^ in ALS (*p* < 0.01). Significant differences remained when comparing controls to patients without dementia: 20.86 ± 4.74 cells/500 μm^2^. Once again, differences may be more pronounced in patients with ALS-FTD at 23 ± 2 cells/500 μm^2^ but patient numbers were too low to permit a statistical analysis (Additional file 1: Tables S4 and S6; Fig. 1k and m).

Tuj-1 only marked the processes of cells and not the neuronal bodies, so a quantitative study could not be completed using this marker.

### Changes in neurogenesis and their correlation with the percentage of cytoplasmic phosphorylated TDP-43

Misallocated (cytoplasmic) phosphorylated TDP-43 is considered a pathologic hallmark of ALS. inclusion body myositis and some forms of FTD [16, 32]. It has been considered also a marker of the propagation of the disease [13, 15, 32]. A correlation analysis for the percentage of cytoplasmic phosphorylated TDP-43 and neurogenesis in the subventricular zone found a positive linear correlation (*p* < 0.05) with a thicker gap layer (Fig. 3a) and a thicker ribbon, but the difference in the latter case was not statistically significant (*p* = 0.057) (Fig. 3b).

Likewise, the correlation was linear and direct for marking of proliferative neural progenitor cells with PCNA (*p* < 0.05) (Fig. 3d) and with Ki-67 (*p* < 0.01) (Fig. 3e); and for glial cells with GFAP (*p* = 0.005) (Fig. 3c). The correlation was also linear and direct for GFAPδ as a marker for pluripotent neural cells (*p* < 0.005) (Fig. 3f).

We found another linear, direct correlation between pTDP-43 and results from 2 different markers of SVZ neuroblasts: PSA-NCAM (*p* < 0.05) (Fig. 3g) and doublecortin (*p* < 0.001) (Fig. 3h).

### Structural organization of the dentate gyrus in patients with ALS

There were no changes in the thickness of the subgranular layer in the dentate gyrus.

### Decrease in proliferative neural progenitor cells in the dentate gyrus

**Fig. 2:**
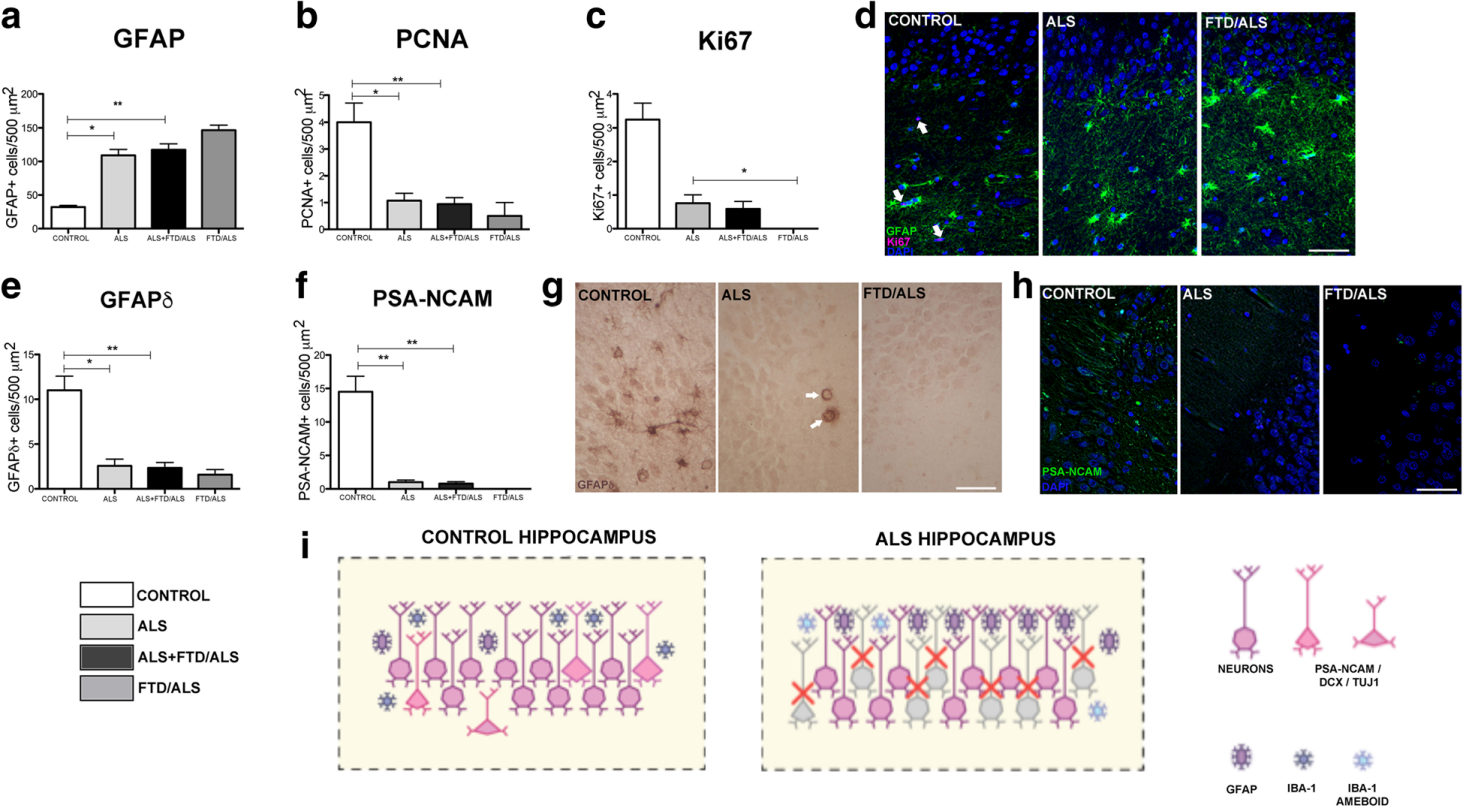
Changes in hippocampal morphology in patients with ALS. **a** GFAP analysis in the hippocampus of ALS patients and controls; protein levels are significantly higher in ALS. **b-c** Quantification of the markers PCNA and Ki-67, which are linked to events in proliferative activity in the hippocampus of patients with ALS; these patients show significant decreases for all markers. **d** Confocal microscope images of the hippocampus in controls and patients with ALS or ALS-FTD. ALS patients show isolated Ki-67–positive cells and increased GFAP marking. **e** Quantification of the expression of GFAPδ, a protein linked to neurogenic processes in the hippocampus; patients with ALS and ALS-FTD show significantly less expression than controls. **f** Graph showing markedly lower expression of PSA-NCAM, a protein linked to neuronal migration, in patients with ALS and ALS-FTD compared to controls. **g** Immunoperoxidase images reveal decreased expression of that marker. GFAPδ cells in patients with ALS were isolated and differed from those observed in controls in that they had fewer processes, a more spherical nucleus (arrow), and were mainly located in the subgranular layer. **h** Images of PSA-NCAM–positive cells in the hippocampus. Tissue samples from controls contained cells with typical elongated processes extending through the granular layer; the ALS group showed isolated, shorter processes, and no processes were observed in the ALS-FTD group. **i** Illustration of the typical morphology of the hippocampus in healthy controls and in patients with ALS showing the principal changes we observed: increase in GFAP-positive cells, neuronal loss, and decrease in markers linked to neuronal migration. Graphs show the mean + standard error (*p < 0.05; **p < 0.01). Scale bars: D, G, H: 40 μm

Analysis of the subgranular zone indicated a decrease in marked proliferative neural progenitor cells. Using PCNA, we found a significantly lower number of marked cells in patients with ALS than in controls: 4 ± 1.41 cells/500 μm^2^ in controls vs 0.94 ± 0.72 cells/500 μm^2^ in ALS patients, (*p* < 0.01). This decrease was also present in cases of ALS without dementia at 1.97 ± 0.73 cells/500 μm^2^, *p* = 0.01). The difference may be even greater in patients with ALS-FTD: 0.5 ± 0.71 cells/500 μm^2^ (Additional file 1: Tables S5 and S7; Fig. 2b). The Ki-67 marker also highlighted a significant decrease in cells in total ALS patients compared to controls: 3.25 ± 0.96 cells/500 μm^2^ in controls vs 0.59 ± 0.66 cells/500 μm^2^ in ALS patients with or without dementia (*p* < 0.01). Control group data for Ki-67 also contrast with results from the subgroup of ALS cases without FTD: 0.75 ± 0.66, (*p* = 0.01) (Additional file 1: Tables S5 and S7; Fig. 2c and d). No cells marked with Ki-67 were visible in the fields examined in patients with ALS-FTD (Fig. 2d).

The study of specimens from the dentate gyrus of the hippocampus using GFAP revealed higher numbers of marked cells in patients with ALS: 32 ± 5.03 cells/500 μm^2^ in controls vs 117 ± 26.24 cells/500 μm^2^ in the total ALS group (*p* < 0.01), 109.1 ± 23.19 cells/500 μm^2^ in ALS without FTD (*p* < 0.01), and 146.5 ± 7.5 cells/500 μm^2^ in ALS-FTD. These cells did not co-localize with other proliferation markers (Additional file 1: Tables S5 and S7; Fig. 2a and d), meaning that the marked cells meet the description of true astrocytes rather than proliferative neural progenitor cells.

### Patients with ALS show lower numbers of GFAPδ neural pluripotent cells (NPCs) in the dentate gyrus

GFAPδ was used in this study as a marker for studying NPCs. We observed a lower number of marked cells in ALS patients compared to controls: 11 ± 3.16 cells/500 μm^2^ in controls vs 2.35 ± 1.8 cells/500 μm^2^ in ALS (*p* < 0.01). This reduction is also significant in ALS cases without dementia at 2.57 ± 0.75 cells/500 μm^2^ (*p* = 0.01) and appears to be more pronounced in ALS-FTD cases: 1.57 ± 0.57 cells/500 μm^2^ (Additional file 1: Tables S5 and S7; Fig. 2e and g). Furthermore, these cells have a different shape with shorter processes (Fig. 2g).

### Fewer neuroblasts observed in the dentate gyrus of ALS patients

We used the markers DCX, Tuj-1 and PSA-NCAM to study neuroblasts in the dentate gyrus. PSA-NCAM yielded fewer marked cells in patients with ALS compared to controls: 14.5 ± 4.99 cells/500 μm^2^ in controls vs 0.79 ± 0.85 cells/500 μm^2^ in all ALS patients (*p* < 0.01). This decrease in marked cells is also significant in ALS cases without dementia at 1.01 ± 0.84 cells/500 μm^2^ (*p* = 0.01) and appears to be more pronounced in ALS-FTD cases: 0.05 ± 0.05 cells/500 μm^2^ (Additional file 1: Tables S5 and S7; Fig. 2f and h).

For dobleucortin and Tuj-1 in ALS-patients we only found some dendrites stained but no neuronal bodies, because of that, a statistical analysis was not perfomed for these two markers.

### Correlation between percentage of pTDP-43 in cytoplasm and the decrease in neurogenesis in the SGZ

**Fig. 3:**
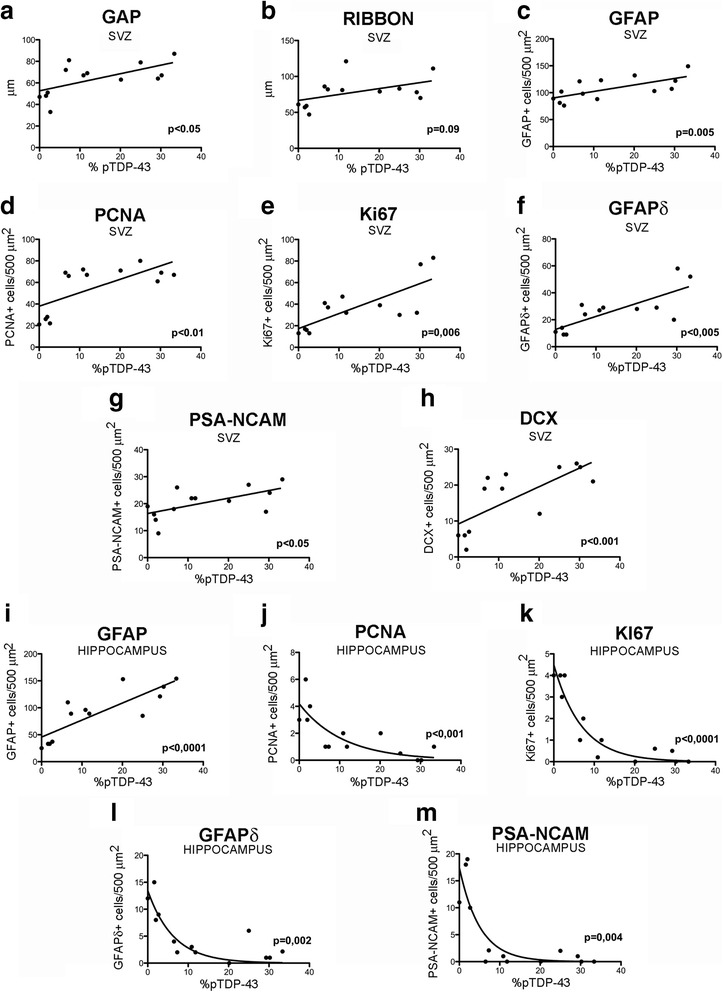
Analysis of the percentage of pTDP-43 and its correlation to markers linked to proliferation and adult neurogenesis in the SVZ and hippocampus in controls and patients with ALS/ALS-FTD. **a** Significant direct linear correlation between the increase in gap layer thickness and the percentage of inclusions positive for pTDP-43. **b** Tendency toward a direct linear correlation between the increase in ribbon layer thickness and percentage of inclusions positive for pTDP-43. **c** Significant direct linear correlation between the increase in GFAP expression and the percentage of pTDAP-43 inclusions. **d** Significant direct linear correlation between the increase in PCNA protein expression in the SVZ and the percentage of inclusions positive for pTDP-43. **e** Significant direct linear correlation between the increase in Ki-67 protein (indicating proliferation in the SVZ) and the percentage of inclusions positive for pTDP-43. **f** Significant direct linear correlation between increased expression of the GFAPδ protein in the SVZ and the percentage of pTDP-43 inclusions. **g** Significant direct linear correlation between increased expression of PSA-NCAM (neuroblast marker) in the SVZ and the percentage of inclusions positive for pTDP-43. **h** Significant direct linear correlation between the increase in the expression of doublecortin (neuroblast marker) and the percentage of inclusions positive for pTDP-43 in the SVZ. **i** Significant direct linear correlation between the increase in the expression of hippocampal GFAP (marker for gliosis) and the percentage of inclusions positive for pTDP-43. **j** The hippocampus shows a significant inverse exponential relationship between the expression of PCNA (proliferation marker) and the percentage of pTDP-43–positive inclusions. **k** Significant inverse exponential relationship between hippocampal expression of the Ki-67 protein (proliferation marker) and the percentage of inclusions positive for pTDP-43. **l** Significant inverse exponential relationship between hippocampal expression of the GFAPδ protein and the percentage of inclusions positive for pTDP-43. **m** The hippocampus displays a significant inverse exponential relationship between the expression of the PSA-NCAM protein (neuroblast marker) and the percentage of pTDP-43 positive inclusions

An analysis of the percentage of cytoplasmic pTDP-43 and the changes observed in the SGZ of the dentate gyrus indicates a linear, direct relationship between the percentage of pTDP-43 and gliosis as marked with GFAPα (*p* < 0.0001, Fig. 3i). There is an exponential and inverse relationship between the percentage of pTDP-43 and the number of cells in the different stages of neurogenesis, as demonstrated by PCNA (*p* < 0.0001) (Fig. 3j); Ki-67 (*p* < 0.0001) (Fig. 3k), for type proliferative neural progenitor cells with GFAPδ (*p* < 0.005) (Fig. 3l). This is also true of neuroblasts observed with PSA-NCAM (type 3/type D3 cells) (*p* < 0.005) (Fig. 3m).

## Discussion

Upon analyzing earlier studies on the state of adult neurogenesis in neurodegenerative diseases, we encounter dissimilar results, although most studies report a decrease in adult neurogenesis where these diseases are present [10, 33–37]. Nevertheless, proliferation may increase during certain early stages of Alzheimer disease [38, 39], although this initial response will vanish as neurogenesis starts to decrease in later stages of the disease [40].Although animal models demonstrate decreased neurogenesis at both niches in Huntington disease [41, 42], human studies seem to indicate increased neurogenesis in the SVZ, with increase in SVZ and increase of GAP layer [36] with no changes in the SGZ [43].

Very few studies have examined adult neurogenesis in ALS. Most of these studies have focused on the neurogenic state of the central canal niche, which seems to exhibit increased neurogenesis preceding symptom onset, according to some authors [20]. Others have reported increased neurogenesis in early but symptomatic stages of the disease [19], while still other studies reported no modifications [44]. Only one study in mice transgenic for the SOD1 mutation has analyzed classic neurogenic niches [18] and found increased neurogenesis in the SVZ with no changes in the SGZ.

Our group previously reported the case of a patient with ALS-FTD who exhibited a significant increase in neurogenesis in the SVZ [21]. That case has been included in this series. There have been no other autopsy studies of neurogenesis.

Our autopsy results show that proliferation in adult neurogenic niches is altered in patients with ALS. We observed increased neurogenesis in the SVZ and reduced neurogenesis in the SGZ of the dentate gyrus. We found some neurogenesis in SGZ on controls that is extremely reduced in ALS patients, however, even in the controls neurogenesis in SGZ is not so important as previously described for some authors [45].This alteration affects all cell types and is present in all ALS cases, whether or not they have associated FTD; however, presence of FTD seems to result in more pronounced changes at both niches (Figs. 1n and 2i). Although the proliferation rate at each of these niches differs greatly (it is 6 times higher in the SVZ than in SGZ [46]), this difference does not offer an explanation of how ALS could give rise to opposite tendencies in neurogenesis at different niches. These increase in proliferation in SVZ has been previously described in Huntington [36] and in non neurodegenerative diseases as stroke [9, 47].

The function of adult neurogenesis is unknown. However, the SVZ in humans is believed to be a site of repair, since cells forming at this niche are able to migrate long distances. The SVZ is also thought to respond to insult since there seems to be increased cell proliferation with migration to the lesion [9]. The role of neurogenesis in the SGZ, in contrast, is not as clear; neurogenesis at this niche has been more often correlated with memory [48]. In any case, a repair function at this niche would only exert a local effect since the neurons formed here do not migrate significantly [48]. The increase in neurogenesis in the SVZ may be understood as a response to the loss of neurons in ALS. However, our results do not clarify what happens to new neurons if they migrate to damaged areas, and they do not show whether they are able to integrate. This increase in neurogenesis in the SVZ was also highlighted by the only study of this niche to be carried out in a transgenic SOD1 animal model [18]. The decrease in neurogenesis in the hippocampus is similar to that described in other neurodegenerative diseases [10]. The study in transgenic SOD1 mice did not report this decrease [18], but we note that this model featured pure motor impairment with no associated cognitive changes.

Another topic for discussion is how neurogenesis may be regulated differently in these 2 niches. One potential explanation is that each niche may be regulated by different factors that would be modified by the disease in different ways. Another possibility is that the same factor would have a different effect on each of the neurogenic niches, although such findings have never been described [33]. Yet another possibility is that the factor responsible for increasing neurogenesis could be delivered to each niche in a different way. For example, if the factor were delivered in CSF, it would be almost unable to act on the SGZ and primarily affect the SVZ by direct contact. Both our group and others have studied the role of CSF in ALS and ALS propagation [26, 49, 50]. Its role in exosome transport to regulate neurogenesis has also been examined [51]. This possibility would also explain the presence of cells whose processes stretch to the CSF in patients with FTD-ALS.

According to our observations, the percentage of cytoplasmic pTDP-43 correlated with the change in neurogenesis in both niches. Other authors have described TDP-43 as a marker of disease diffusion and progression that may even be useful for establishing different stages of the disease [38]. This correlation might therefore indicate a relationship between disease progression and changes in neurogenesis, although our study does not permit us to state this as a conclusion.

## Conclusions

Our observations show that adult neurogenesis is altered in patients with ALS: it increases in the SVZ and decreases in the SGZ (Figs. 1n and 2i). Although it is an interesting fact, this study does not let us determine whether this increase in the SVZ has a true impact on the disease and if the new neurons are able to migrate to damaged areas, integrate, and become functional by activating a primitive neural repair response similar to that occurring in other species [1, 46, 52].

## Additional file

Additional file 1: Table S1. Antibodies used in the immunohistochemical study. **Table S2.** Summary of patient characteristics. **Table S3.** Immunohistochemical studies used in ALS diagnosis. Values for TDP-43 and ubiquitin are expressed in inclusions per field; %pTDP-43 represents the percentage of phosphorylated TDP inclusions out of the total. **Table S4.** Description of neurogenesis patient to patient. **Table S5.** Neurogenesis findings in the subgranular zone of the hippocampal dentate gyrus, by patient. **Table S6.** Summary of results in the SVZ. **Table S7.** Summary of results in the hippocampus. (DOC 302 kb)

## Abbreviations

ALS: Amyotrophic Lateral Sclerosis
CSF: Cerebrospinal fluid
DAPI: 4,6 diamino-2-phenylindole
DCX: Doublecortin
FTD: Frontotemporal dementia
GFAP: Glial Fibillary Acidic Protein
PBS: Phosphate buffered saline
PCNA: Proliferating cell nuclear antigen
PNC: Pluripotent neural cells
pTDP-43: Phosphorylated Transactivator regulatory DNA-binding protein 43
RMS: Rostral Migratory Stream
SD: Standard deviation
SGZ: Subgranular zone
SOD1: Superoxide dismutase1
SVZ: Subventricular zone
TUJ1: Neuron-specific class III beta-tubulin
