# Supplementary material for: Amyotrophic lateral sclerosis modifies progenitor neural proliferation in adult classic neurogenic brain niches
Source: BMC Neurol. 2017 Sep 6;17:173. doi: 10.1186/s12883-017-0956-5 (PMC5585932; doi:10.1186/s12883-017-0956-5)
Supplement: Additional file 1: Table S1. — Antibodies used in the immunohistochemical study. Table S2. Summary of patient characteristics. Table S3. Immunohistochemical studies used in ALS diagnosis. Values for TDP-43 and ubiquitin are expressed in inclusions per field; %pTDP-43 represents the percentage of phosphorylated TDP inclusions out of the total. Table S4. Description of neurogenesis patient to patient. Table S5. Neurogenesis findings in the subgranular zone of the hippocampal dentate gyrus, by patient. Table S6. Summary of results in the SVZ. Table S7. Summary of results in the hippocampus. (DOC 302 kb) [file 12883_2017_956_MOESM1_ESM.doc]

Additional file 1

Table S1 Antibodies used in the immunohistochemical study

| Primary antibodies | | | | | | | | |
| --- | --- | --- | --- | --- | --- | --- | --- | --- |
| Antibody | Host | Dilution | Source/catalog | | | Immunogen | | Description |
| Ubiquitin | Rabbit | 1:100 | Abcam/  ab7780 | | | Complete recombinant protein (human) | | Specifically recognizes ubiquitinated cytoplasmic inclusions. |
| TDP43 | Rabbit | 1:100 | Cell Signaling Technology/ #3449 | | | Synthetic peptide (conjugated to KLH) corresponding to residues surrounding Ala260 of TDP43 | | TDP43 (TAR DNA-binding protein 43) related to transcriptional regulation of exon splicing |
| PCNA | Rat | 1:100 | Abcam A29 | | | Proliferating cell nuclear antigen | | Proliferation marker |
| Ki-67 | Mouse | 1:100 | Dako/  M7240 | | | Recombinant human peptide corresponding to a cDNA Ki-67 fragment of 1002bp | | Cell proliferation marker (all phases of the cell cycle except G0) |
| S100 | Rabbit | 1:1 | Immunostar/  22520 | | | Protein S100 isolated from the bovine brain | | Ependymal and glial marker in the central nervous system |
| PSA-NCAM | Mouse | 1:400 | AbCys/  AbC0019  Clone 355 | | | Meningococcus group B (strain 355) | | Marker of migrating neuroblasts |
| Tuj1 | Chicken | 1:500 | Millipore / AB9354 | | | Class III β-tubulin | | Early neuronal marker |
| DCX | Goat | 1:500 | Santa Cruz SC-8066 | | | Protein associated with microtubules in neuroblasts | | Neuroblast marker |
| GFAP-δ | Rabbit | 1:500 | Millipore/  AB9598 | | | Glial fibrillary acid protein | | Exclusively marks the δ isoform of astrocytes. Does not recognize GFAPα astrocytes. |
| GFAP-α | Rabbit | 1:600 | Dako/  Z0334 | | | Glial fibrillary acid protein | | Astrocyte marker |
| GFAP-α | Mouse | 1:600 | Sigma/  C9205  Clone G-A-5 Cy3 | | | Glial fibrillary acid protein | | Astrocyte marker |
| IBA1 | Goat | 1:200 | Wako  019-19741 | | | Calcium-dependent protein coded by the AIF1 gene | | Marker for microglia and macrophages |
| Secondary antibodies | | | | | | | | |
| Antibody | | | | Host | Dilution | | Source/catalog | |
| Anti-rabbit IgG peroxidase | | | | Goat | 1:200 | | Vector Labs/ PI-1000 | |
| Alexa Fluor 488 anti Chicken 1gG | | | | Goat | 1:500 | | Invitrogen/ A11039 | |
| Alexa Fluor 555 anti Mouse 1gG | | | | Goat | 1:500 | | Invitrogen/ A21424 | |
| Alexa Fluor 555 anti Mouse 1gM | | | | Goat | 1:500 | | Invitrogen/ A21426 | |
| Alexa Fluor 488 anti Mouse 1gG | | | | Goat | 1:500 | | Invitrogen/ A11029 | |
| Alexa Fluor 647 anti Rabbit 1gG | | | | Goat | 1:500 | | Invitrogen/ A21245 | |

Table S2 Summary of patient characteristics

| Patient number | Form of onset | Range og age at diagnosis | El Escorial criteria | Time symptom onset–diagnosis (months) | Associated disease | NIV | Riluzole | Survival from onset (months) | Survival from diagnosis | Dementia | Cause of death |
| --- | --- | --- | --- | --- | --- | --- | --- | --- | --- | --- | --- |
| Patient 1 | Bulbar | 60-65 | Probable | 23 | HTA | No | No | 24 | 1 | Yes | Respiratory insuffiency |
| Patient 2 | Bulbar | 65-70 | Probable | 38 | No | No | No | 48 | 10 | Yes | Respiratory insuffiency |
| Patient 3 | Spinal | 30-35 | Definitive | 6 | Crohn’s disease | Yes | Yes | 64 | 58 | No | Respiratory insuffiency |
| Patient 4 | Spinal | 55-60 | Definitive | 11 | Smoking | Yes | Yes | 18 | 7 | No | Respiratory insuffiency |
| Patient 5 | Spinal | 45-50 | Definitive | 6 | No | Yes | Yes | 17 | 11 | No | Respiratory insuffiency |
| Patient 6 | Bulbar | 85-90 | Probable | 2 | No | No | No | 3 | 1 | No | Respiratory insuffiency |
| Patient 7 | Bulbar | 80-81 | Definitive | 11 | No | Yes | Yes | 14 | 3 | No | Respiratory insuffiency |
| Patient 8 | Bulbar | 70-75 | Probable | 13 | Essential tremor | Yes | Yes | 26 | 13 | No | Respiratory infection |
| Patient 9 | Spinal | 65-70 | Probable | 2 | Myasthenia gravis. Thymoma. Atrial fibrillation | Yes | Yes | 10 | 8 | No | Respiratory insuffiency |

F: female M: Male

|  | EMG | Pyramidalism | Fasciculations | ALS-Health State Scale |
| --- | --- | --- | --- | --- |
| Patient 1 | Fasciculations, denervation and positive waves in bulbar, and lumbar region | From mentonian | Quadriceps,calfs | At death III |
| Patient 2 | Fasciculations, denervation and positive waves in bulbar, cervical and lumbar region | From C5 | Tongue, calfs | AT death III |
| Patient 3 | Fasciculations, denervation and positive waves in bulbar, cervical thorciccand lumbar region | From C5 | Facial, tongue, pectoral, supraspinous, supinator, quadriceps, calf | AT death IV |
| Patient 4 | Fasciculations, denervation and positive waves in bulbar, cervical thoraciccand lumbar region | From mentonian | Tongue, deltoids, pectorals, quadriceps, calf | At death IV |
| Patient 5 | Fasciculations, denervation and positive waves in bulbar, cervical thoraciccand lumbar region | From mentonian | Tongue, deltoids, pectorals, supraspinatus quadriceps, calf | At death IV |
| Patient 6 | Fasciculations, denervation and positive waves in bulbar, and lumbar region | From C7 | Deltoids, supra and infraspinatus | At death IV |
| Patient 7 | Fasciculations, denervation and positive waves in bulbar cervical, thoracic and lumbarregion | From mentonian | Tongue, deltoids, supinator, pectoral, quadriceps calf | At death III |
| Patient 8 | Fasciculations, denervation and positive waves in bulbar, and cervical region | From mentonian | Tongue and deltoids | At death III |
| Patient 9 | Fasciculations, denervation and positive waves in bulbar, and lumbar region | From C4 | Tongue, calfs, quadriceps | At death III |

Table S3 Immunohistochemical studies used in ALS diagnosis. Values for TDP-43 and ubiquitin are expressed in inclusions per field; %pTDP-43 represents the percentage of phosphorylated TDP inclusions out of the total.

|  | TDP-43 | %pTDP-43 (cytoplasmic) | Ubiquitin |
| --- | --- | --- | --- |
| Patient 1 (ALS-FTD) | 15 | 33.34 | 9 |
| Patient 2 (ALS-FTD) | 19 | 30.21 | 8 |
| Patient 3 | 12 | 20.15 | 6 |
| Patient 4 | 13 | 11.8 | 9 |
| Patient 5 | 14 | 6.5 | 9 |
| Patient 6 | 17 | 29.3 | 4 |
| Patient 7 | 16 | 25 | 5 |
| Patient 8 | 7 | 7.31 | 7 |
| Patient 9 | 13.4 | 10.87 | 12 |
| CONTROL 1 | 14 | 2 | 1 |
| CONTROL 2 | 11 | 0 | 0 |
| CONTROL 3 | 8 | 1.61 | 1 |
| CONTROL 4 | 14 | 2.67 | 0 |

Table S4 Description of neurogenesis patient to patient

| Neurogenesis in SVZ | | | | | | | | |
| --- | --- | --- | --- | --- | --- | --- | --- | --- |
| Patients | Morphological changes  In SVZ | | Changes in histochemical markers in SVZ | | | | | |
|  | Gap layer (μm2) | Ribbon layer (μm2) | Cell proliferation | | NPCs | Astrocytes | Neurogenesis / Neuroblasts | |
|  |  |  | PCNA | Ki-67 | GFAPδ | GFAP | PSA-NCAM | DCX |
| Patient 1 (ALS + FTD) | 87 ± 19 | 111 ± 22 | 67 ± 7 | 83 ± 11 | 52 ± 3 | 149 ± 29 | 29 ± 3 | 21.00 |
| Patient 2 (ALS-FTD) | 67 ± 6 | 70 ± 9 | 69 ± 4 | 77 ± 5 | 58 ± 8 | 122 ± 9 | 24 ± 2 | 25.00 |
| Patient 3 | 63 ± 7 | 79 ± 12 | 71 ± 6 | 39 ± 3 | 28 ± 8 | 132 ± 9 | 21 ± 9 | 12.00 |
| Patient 4 | 69 ± 6 | 121 ± 21 | 67 ± 2 | 32 ± 7 | 29 ± 7 | 123 ± 10 | 22 ± 3 | 23.00 |
| Patient 5 | 72 ± 6 | 86 ± 12 | 69 ± 6 | 41 ± 8 | 31 ± 2 | 121 ± 9 | 18 ± 4 | 19.00 |
| Patient 6 | 64 ± 7 | 78 ± 7 | 61 ± 6 | 32 ± 4 | 20 ± 3 | 107 ± 13 | 17 ± 3 | 26.00 |
| Patient 7 | 79 ± 8 | 83 ± 2 | 80 ± 1 | 30 ± 6 | 29 ± 7 | 103 ± 13 | 27 ± 1 | 25.00 |
| Patient 8 | 81 ± 6 | 82 ± 7 | 66 ± 9 | 37 ± 4 | 24 ± 13 | 98 ± 12 | 26 ± 1 | 22.00 |
| Patient 9 | 67 ± 9 | 81 ± 7 | 72 ± 4 | 47 ± 3 | 27 ± 4 | 88 ± 11 | 22 ± 3 | 19.00 |
| Control 1 | 51 ± 9 | 59 ± 10 | 28 ± 7 | 16 ± 8 | 9 ± 3 | 102 ± 12 | 14 ± 3 | 2.00 |
| Control 2 | 47 ± 3 | 61 ± 7 | 21 ± 1 | 13 ± 7 | 11 ± 2 | 89 ± 11 | 19 ± 1 | 6.00 |
| Control 3 | 48 ± 7 | 57 ± 9 | 26 ± 5 | 17 ± 6 | 14 ± 1 | 81 ± 13 | 16 ± 2 | 6.00 |
| Control 4 | 33 ± 3 | 47 ± 3 | 22 ± 1 | 13 ± 5 | 9 ± 3 | 76 ± 11 | 9 ± 3 | 7.00 |

is observed in the SVZ, by patient

Table S5 Neurogenesis findings in the subgranular zone of the hippocampal dentate gyrus, by patient

| Summary of results | | | | | | | |
| --- | --- | --- | --- | --- | --- | --- | --- |
| Patients |  | Change in immunohistochemical markers in hippocampus | | | | | |
|  |  | Cell proliferation | NPCs | Astrocytes | Neurogenesis /  neuroblasts | | Macrophages/ morphology |
|  | PCNA | Ki-67 | GFAPδ | GFAP | PSA-NCAM | DCX | IBA-1 |
| Patient 1 (ALS-FTD) | 1.00 | 0.00 | 2.15 | 154.00 | 0.00 | + | 18  ameboid |
| Patient 2 (ALS-FTD) | 0.00 | 0.00 | 1.00 | 139.00 | 0.00 | + | 16  ameboid |
| Patient 3 (ALS) | 2.00 | 0.00 | 0.00 | 153.00 | 0.00 | + | 19  ameboid |
| Patient 4 (ALS) | 1.00 | 1.00 | 2.00 | 89.00 | 0.00 | + | 18  ameboid |
| Patient 5 (ALS) | 1.00 | 1.00 | 4.00 | 110.00 | 0.00 | + | 25  ameboid |
| Patient 6 (ALS) | 0.00 | 0.50 | 1.00 | 121.00 | 1.00 | + | 15  ameboid |
| Patient 7 (ALS) | 0.50 | 0.60 | 6.00 | 85.00 | 2.00 | + | 17  ameboid |
| Patient 8 (ALS) | 1.00 | 2.00 | 2.00 | 89.00 | 2.10 | + | 18  ameboid |
| Patient 9 (ALS) | 2.00 | 0.20 | 3.00 | 96.00 | 1.00 | + | 17  ameboid |
| Control 1 | 3.00 | 3.00 | 8.00 | 33.00 | 19.00 | ++ | 27  branching |
| Control 2 | 3.00 | 4.00 | 12.00 | 25.00 | 11.00 | ++ | 33  branching |
| Control 3 | 6.00 | 4.00 | 15.00 | 33.00 | 18.00 | + | 25  branching |
| Control 4 | 4.00 | 4.00 | 9.00 | 37.00 | 10.00 | + | 38  branching |

Table S6 Summary of results in the SVZ

|  |  | Control | ALS total | p | ALS without FTD | p |
| --- | --- | --- | --- | --- | --- | --- |
| Structure | Gap layer | 44.75 ± 8.02 | 73.78 ± 3.57 | 0.007 | 71.14 ± 3.99 | 0.006 |
|  | Ribbon layer | 53.5 ± 5.51 | 89.89 ± 16.09 | 0.003 | 87.29 ± 13.28 | 0.006 |
|  | GFAPα | 87 ± 11.34 | 119 ± 18.97 | 0.001 | 112.3 ± 4.66 | 0.002 |
| Proliferation | PCNA | 24.25 ± 3.3 | 73.22 ± 9.83 | 0.002 | 69 ± 1.86 | 0.003 |
|  | Ki-67 | 14.75 ± 2.06 | 46.89 ± 17.40 | 0.007 | 38.43 ± 1.99 | 0.01 |
| NPCs | GFAPδ | 10.75 ± 2.36 | 35.89 ± 14.49 | 0.007 | 29 ± 2.05 | 0.01 |
| Neuroblasts | PSA-NCAM | 6.5 ± 2.51 | 19.78 ± 1.63 | 0.007 | 18 ± 3.51 | 0.01 |
|  | DCX | 5.25 ± 2.22 | 21.33 ± 1.44 | 0.01 | 20.86 ± 4.74 | 0.001 |

Significance level is p < 0.05; ns = not significant

Table S7 Summary of results in the hippocampus

|  |  | Control | ALS total | p | ALS without FTD | p |
| --- | --- | --- | --- | --- | --- | --- |
| Structure | GFAP in astrocytes | 32 ± 5.03 | 117.40 ± 26.24 | 0.007 | 109 ± 23.19 | 0.01 |
|  | IBA1 in microglia | 30.75 ± 5.91  (branching) | 0.94 ± 3.7  (ameboid) | 0.009 | 18.4 ± 4.18  (ameboid) | 0.01 |
| Proliferation | PCNA | 4 ± 1.41 | 0.94 ± 0.72 | 0.006 | 1.97 ± 0.73 | 0.01 |
|  | Ki-67 | 3.25 ± 0.96 | 0.59 ± 0.66 | 0.008 | 0.75 ± 0.66 | 0.01 |
| NPCs | GFAPδ | 11 ± 3.16 | 2.35 ± 1.8 | 0.007 | 2.57 ± 0.75 | 0.004 |
| Neuroblasts | PSA-NCAM | 14.5 ± 4.66 | 0.79 ± 0.85 | 0.006 | 1.01 ± 0.84 | 0.01 |

p < 0.05 considered significant
